# Population pharmacokinetic modeling and Monte Carlo simulation to optimize meropenem dosing in patients with severe postoperative infections

**DOI:** 10.3389/fphar.2026.1778552

**Published:** 2026-04-09

**Authors:** Yang Zou, Jianke Ren, Haibo Lei, Guanghui Chen, Chenyan Li, Xiangping He, Yixiang Hu, Xiang Liu

**Affiliations:** 1 Department of Clinical Pharmacy, The Central Hospital of Xiangtan (The affiliated Hospital of Hunan University), Xiangtan, China; 2 Department of Nephrology and Rheumatology, The Central Hospital of Xiangtan (The affiliated Hospital of Hunan University), Xiangtan, China; 3 General Intensive Care Unit, The Central Hospital of Xiangtan (The affiliated Hospital of Hunan University), Xiangtan, China; 4 State Key Laboratory of Chemo/Bio-Sensing and Chemometrics, School of Biomedical Sciences, Hunan University, Changsha, China

**Keywords:** meropenem, Monte Carlo simulation, PK/PD, population pharmacokinetics, postoperative infections, therapeutic drug monitoring

## Abstract

**Background:**

Meropenem is commonly used to treat severe postoperative infections. However, substantial pharmacokinetic variability among surgical patients makes dose optimization challenging. Population pharmacokinetic (PopPK) modeling integrated with pharmacokinetic/pharmacodynamic (PK/PD) simulations may help support individualized dosing in this setting.

**Methods:**

Adult patients with severe postoperative infections receiving meropenem were prospectively enrolled for therapeutic drug monitoring. Plasma meropenem concentrations were determined using a validated high-performance liquid chromatography (HPLC) assay. A nonlinear mixed-effects modeling approach was used to characterize meropenem pharmacokinetics and to explore potential covariates. Monte Carlo simulations (MCS) were performed to estimate the probability of target attainment (PTA) across different renal function categories and minimum inhibitory concentrations (MICs), using PK/PD targets of 40% fT > MIC, 100% fT > MIC, 50% fT > 4×MIC, and 100% fT > 4×MIC.

**Results:**

44 patients with 135 concentration measurements were analyzed. Meropenem pharmacokinetics were described by a one-compartment model with linear elimination. The typical population clearance (CL) and apparent volume of distribution (Vc) were 6.472 L/h and 26.69 L, respectively. Postoperative creatinine clearance (CRCL) was the only significant covariate affecting CL. Patients with augmented renal clearance or elevated MIC values may require intensified dosing strategies, whereas dose reduction is generally required in patients with renal impairment. For MIC ≤1 mg/L, 500 mg every 8 h achieved adequate PTA across most renal strata. At MIC 4 mg/L, intensified regimens (e.g., 1 g every 6–8 h or prolonged infusion) were required depending on renal function. At MIC 8 mg/L, even high-dose regimens were insufficient to achieve stringent PK/PD targets.

**Conclusion:**

Postoperative renal function is a major determinant of meropenem exposure in patients with severe postoperative infections. PopPK-guided dosing combined with Monte Carlo simulation provides a practical framework for optimizing meropenem therapy and facilitating individualized antimicrobial treatment. Selecting dosing regimens according to renal function and MIC may improve the therapeutic effectiveness of meropenem in this population.

## 1 Introduction

Postoperative infections, including surgical site infections (SSIs) and other infection-related complications, remain major contributors to prolonged hospitalization, increased medical costs, poor clinical outcomes, and an elevated risk of disease recurrence (Jiang et al., 2020; Groenen et al., 2024). The reported incidence of postoperative infections in general surgery ranges from 4.29% to 12.3%, with abdominal and pulmonary procedures accounting for the majority of cases (Tan et al., 2020). Between 2018 and 2020, the overall incidence of SSIs following colorectal surgery in China was 5.6%, which is lower than the reported rate of postoperative abdominal infections in Japan (13.7%) (Maruyama et al., 2020). Notably, patients with pre-existing intra-abdominal infections prior to surgery are at a markedly increased risk of developing postoperative sepsis or septic shock, making this condition the second most common cause of postoperative infection after pulmonary infections (Huston et al., 2024).

Meropenem is a broad-spectrum β-lactam antibiotic with potent and stable bactericidal activity against Gram-negative and Gram-positive bacteria as well as anaerobic pathogens, and it demonstrates favorable tissue penetration (Logan et al., 2025; Singh et al., 2023; Ullah et al., 2023). Consequently, it is widely used for the treatment of severe postoperative infections and is frequently administered as empirical therapy in patients with severe sepsis or septic shock in intensive care units (ICUs), where its clinical efficacy has been well documented (Liao et al., 2026). As a time-dependent antimicrobial agent, the *in vivo* efficacy of meropenem is primarily determined by the proportion of the dosing interval during which the free plasma concentration exceeds the minimum inhibitory concentration (%fT > MIC), a parameter that is commonly used as the key PK/PD target (Lauro Vieira et al., 2018; Steffens et al., 2021). %fT > MIC is closely influenced by factors such as drug half-life, plasma exposure, and pathogen susceptibility. However, the optimal PK/PD target for meropenem has not yet been conclusively defined. While a %fT > MIC of approximately 40% is generally considered sufficient to achieve favorable clinical outcomes in patients with mild to moderate infections, critically ill patients often require much more aggressive targets, such as 100% fT > MIC or even 100% fT > 4–5×MIC, to maximize clinical cure rates and bacterial eradication (Dhillon, 2018). In recent years, numerous studies have focused on individualized meropenem dosing strategies across diverse pathophysiological conditions and special populations, including patients with acute kidney injury, those receiving continuous renal replacement therapy (CRRT) or extracorporeal membrane oxygenation (ECMO), critically ill patients, the elderly, infants, and individuals with obesity (Jorgensen and Rybak, 2018; Marín et al., 2023; Jin et al., 2025). Other investigations have addressed variability related to infection sites (e.g., central nervous system, pulmonary, and bloodstream infections) and multidrug-resistant pathogens, such as carbapenem-resistant *Klebsiella pneumoniae* and carbapenem-resistant *Acinetobacter* baumannii (Pascale et al., 2019; Bradley et al., 2025; Farrington et al., 2024). These studies consistently demonstrate substantial interindividual variability in pharmacokinetic parameters, driven by multiple covariates, which substantially complicates dose optimization. Severe postoperative infections develop in the setting of surgical trauma, hemodynamic instability, large-volume fluid resuscitation, and dynamic changes in renal function (Steffens et al., 2021). Compared with non-surgical critically ill populations, postoperative patients may exhibit transient augmented renal clearance or fluctuating creatinine clearance, resulting in unpredictable meropenem exposure. Previous work by investigators in Pakistan characterized the pharmacokinetics of meropenem in postoperative critically ill patients and identified CRCL as a major determinant of drug clearance, reporting a population clearance value of 12.2 L/h (Wicha et al., 2024). This finding contrasts with results from a separate study conducted in ICU patients with severe sepsis or early septic shock who did not undergo surgery, in whom meropenem clearance was estimated at 7.82 L/h (Jaruratanasirikul et al., 2015; Al-Shaer et al., 2020). These discrepancies suggest that surgical stress and surgery-related physiological alterations may significantly affect meropenem disposition. Despite extensive pharmacokinetic research in critically ill cohorts, data specifically focusing on severe postoperative infections remain limited. Therefore, dose optimization strategies tailored to this population are urgently needed. The present study aimed to characterize the population pharmacokinetic (PopPK) profile of meropenem in patients with severe postoperative infections and to identify physiological factors influencing pharmacokinetic parameters by developing a PopPK model and performing Monte Carlo simulations (MCS). In addition, the study sought to provide dosing recommendations stratified by renal function to support individualized therapy in this patient population.

## 2 Materials and methods

### 2.1 Study population and ethical approval

Patients receiving meropenem for the treatment of postoperative infections were prospectively enrolled in the general intensive care unit (ICU) of our hospital between March 2023 and January 2025, during which therapeutic drug monitoring (TDM) was conducted. The inclusion criteria were as follows: (1) postoperative infection requiring meropenem therapy; (2) continuous meropenem administration for more than 3 days; (3) availability of at least one steady-state plasma concentration; and (4) age >18 years. The exclusion criteria were: (1) pregnancy or lactation; (2) documented allergy to carbapenem antibiotics; (3) concomitant use of sodium valproate; and (4) preoperative infection treated with meropenem. This study was approved by the Institutional Ethics Committee (approval No. 2023–05–015), and written informed consent was obtained from all enrolled patients.

### 2.2 Data collection

Clinical data were obtained from the electronic medical record system, including: (1) demographic characteristics (sex, age, and body weight); (2) infection-related diagnoses; (3) laboratory parameters measured 1 day before and at the initiation of meropenem therapy, including complete blood count, liver and renal function tests, procalcitonin (PCT), and C-reactive protein (CRP); (4) surgical information; (5) dialysis status; (6) details of meropenem therapy, including dose, dosing frequency, route of administration, infusion duration, treatment course, and concomitant medications; and (7) clinical response to antimicrobial treatment.

### 2.3 Dosing regimen and blood sampling

Patients received standard meropenem therapy at a dose of 1.0 g every 8 h (q8h) administered as a prolonged intravenous infusion over 2 h or 2.5 h. Dose adjustment was permitted at the discretion of the treating physician based on TDM results, and repeat concentration measurements were performed when regimen modifications occurred. Blood samples were collected at steady state following at least four consecutive doses. Venous blood samples (2 mL) were obtained using EDTA-K2 vacuum tubes at 30 min before dosing and at 2 h (or 2.5 h), 4 h, and 6 h after the start of infusion. Approximately three sampling time points were randomly collected per patient. Samples were immediately stored at 4 °C and transported to the TDM laboratory within 2 h.

### 2.4 Measurement of meropenem plasma concentrations

Blood samples were centrifuged at 12,000 rpm for 8 min, and the plasma was separated and stored at −80 °C until analysis. Meropenem concentrations were quantified using a validated high-performance liquid chromatography (HPLC) method. The chromatographic conditions were as follows: Thermo Hypersil Gold column (150 × 4.6 mm, 5 μm); mobile phase consisting of 20 mM potassium dihydrogen phosphate and acetonitrile (0.15:0.85, v/v); detection wavelength of 299 nm; flow rate of 0.6 mL/min; column temperature maintained at 30 °C; and an injection volume of 40 μL. Calibration curves were established over a concentration range of 0.5–80.0 μg/mL using weighted linear regression and demonstrated good linearity, with correlation coefficients (*R* ^2^) exceeding 0.99. The analytical accuracy ranged from 100.21% to 111.00%, and the extraction recovery ranged from 83.13% to 88.04%. The intra-batch and inter-batch precision ranges were 4.95%–10.07% and 4.94%–9.07%, respectively. Accuracy was calculated as (measured concentration/nominal concentration) × 100% using quality control samples at low, medium, and high concentration levels. Precision was expressed as the relative standard deviation (RSD%). Both intra-batch and inter-batch validation were conducted in accordance with standard bioanalytical method validation guidelines.

### 2.5 Population pharmacokinetic model development and validation

PopPK analysis was performed using Phoenix NLME software (version 8.1) with a nonlinear mixed-effects modeling approach. Pharmacokinetic parameters were estimated using the first-order conditional estimation with extended least squares (FOCE-ELS) method. Interindividual variability was described using an exponential error model. Residual unexplained variability was assessed using additive, proportional, and combined error models. Model selection was guided by changes in the objective function value (OFV), the Akaike information criterion (AIC), and goodness-of-fit diagnostics. Both one-compartment and two-compartment models with linear elimination were evaluated, and the structural base model was selected based on OFV reduction and goodness-of-fit plots.

Potential covariates, including sex, age, body weight, white blood cell count, platelet count, hemoglobin, neutrophil percentage, alanine aminotransferase, aspartate aminotransferase, total bilirubin, albumin, CRCL, postoperative CRCL, procalcitonin, C-reactive protein, and hemodialysis status, were individually incorporated into the base model using a stepwise covariate modeling (SCM) approach based on the extended least squares principle. Changes in −2 log likelihood (−2LL) were used to assess statistical significance. A stringent criterion was applied, with a forward inclusion threshold of α = 0.05 (OFV decrease >3.84) and a backward elimination threshold of α = 0.001 (OFV increase >10.83). Population typical values of pharmacokinetic parameters and significant covariates influencing parameter variability were identified.

Model performance was evaluated using diagnostic plots, including observed versus population-predicted concentrations, observed versus individual-predicted concentrations, conditional weighted residuals (CWRES) versus population predictions, and CWRES versus time after dosing. Internal validation was conducted using predictive checks visual predictive checks (pcVPCs) and bootstrap analysis. For pcVPCs, 1,000 simulations were performed, and the agreement between observed and simulated concentrations was assessed by comparing the median and 5th and 95th percentiles over time. Model stability was confirmed by the consistency between the median parameter estimates from 500 bootstrap replicates and those from the original model, as well as by the fact that the 95% confidence intervals (2.5%–97.5%) included the final model parameter values.

### 2.6 Monte Carlo simulation

Monte Carlo simulations were conducted using Phoenix NLME to evaluate various dosing regimens and infusion strategies. A total of 500 simulations were performed for each scenario. Dosing regimens included single doses of 500 mg, 1 g, or 2 g administered every 6 h (q6h), every 8 h (q8h), or every 12 h (q12 h), with infusion durations of 30 min or 2 h. Simulations were stratified by the meropenem package insert and renal function based on creatinine clearance calculated by Cockcroft-Gault equation (<10 mL/min, 10–25 mL/min, 26–50 mL/min, and 51–90 mL/min and >90 mL/min). Simulations for CRCL <10 mL/min were exploratory and should be interpreted cautiously due to the absence of observed data in this renal function range. Given that protein binding of meropenem is approximately 2%, total plasma concentrations were assumed to closely approximate free drug concentrations in the simulations. PK/PD targets were defined as 40% fT > MIC, 100% fT > MIC, 50% fT > 4×MIC or 100% fT > 4×MIC. Due to limited availability of bacterial culture and susceptibility data, MIC values were selected according to the 2025 CLSI susceptibility breakpoints for common ICU pathogens, including *Enterobacterales*, *Pseudomonas aeruginosa*, and *Acinetobacter baumannii*, using MIC values of 1, 2, 4, and 8 mg/L for analysis. PTA was calculated for each dosing regimen at specific MIC values, and a PTA ≥90% was considered indicative of an optimal empirical dosing strategy.

## 3 Results

### 3.1 Patient characteristics

A total of 44 patients were included in this study, providing 135 plasma meropenem concentration measurements. Among them, 31 patients were male (70%). The mean age and body weight were 68.5 ± 14.8 years (range: 20.0–91.0) and 56.7 ± 9.04 kg (range: 40.0–81.0), respectively. The mean CRCL was 54.3 ± 29.6 mL/min (range: 11.9–136). Postoperatively, patients generally exhibited a negative fluid balance and markedly elevated infection-related biomarkers. Intra-abdominal infection combined with pulmonary infection was the most common presentation, accounting for 47.73% of cases. Detailed baseline characteristics are summarized in Table 1.

**TABLE 1:** Patient demographics, laboratory characteristics and sampling characteristics.

Patient demographic and laboratory data	Value
Gender (male), N (%)	31 (70.45%)
Gender (female), N (%)	13 (29.55%)
Age (years)	66.0 [20.0, 83.0]^b^
Weight (kg)	58.0 [45.0,81.0]^b^
Type of infection, N (%)	
Intra-abdominal infection	9 (20.45%)
Hospital-acquired pneumonia	10 (22.73%)
Intra-abdominal infection + hospital-acquired pneumonia	21 (47.73%)
Other	4 (9.09%)
Critically ill patients, N (%)	41 (93.18%)
Hemodialysis, N (%)	7 (15.91%)
Preoperative-24 h fluid intake (mL)	2,170 [30.3, 4,250]^b^
Preoperative-24 h fluid out (mL)	1,560 [29.6, 4,450]^b^
Postoperative-24 h fluid intake (mL)	1,670 [871, 2,800]^b^
Postoperative-24 h fluid out (mL)	1,690 [650, 4,420]^b^
The laboratory data	
White blood cells (10^^9^/L)	10.6 [3.75, 27.9]^b^
Red blood cells (10^^12^/L)	3.55 [2.56, 5.48]^b^
Platelets (10^^9^/L)	206 [61.0, 528]^b^
Hemoglobin (g/L)	105 [73.0, 166]^b^
Neutrophil percentage (%)	88.6 [74.9, 97.0]^b^
Alanine aminotransferase (U/L)	13.1 [3.30, 206]^b^
Aspartate aminotransferase (U/L)	28.1 [8.50, 206]^b^
Albumin (g/L)	31.9 [17.7, 44.4]^b^
Total bilirubin (μmol/L)	12.7 [2.30, 78.2]^b^
Urea nitrogen (mmol/L)	8.45 [2.30, 19.5]^b^
Uric acid (μmol/L)	267 [67.0, 507]^b^
Creatinine (μmol/L)	95.0 [37.0, 401]^b^
Creatinine clearances (mL/min)^a^	47.7 [11.9, 136]^b^
Inflammatory indicators	
Procalcitonin (μg/L)	9.30 [0.270, 100]^b^
C-reactive protein (mg/L)	88.4 [0.530, 339]^b^
Sampling characteristics	
Dosage regimen	1.0 g q8h with infusion durations of 2 h or 2.5 h
Meropenem concentration (mg/L)	16.183 [0.477,71.507]^b^

^a^ Represents creatinine clearances was calculated by Cockcroft-Gault equation.

^b^ Mean (minimum, maximum).

### 3.2 Population pharmacokinetic modeling

After evaluating multiple residual error models and comprehensively comparing diagnostic plots and OFV, a one-compartment model with linear elimination was selected as the structural base model. The final model incorporated exponential interindividual variability with a proportional residual error model. During correlation analysis, for covariates with significant collinearity (absolute correlation coefficient ≥0.6), only one variable was retained for subsequent covariate testing. Through stepwise covariate modeling, postoperative creatinine clearance was ultimately identified as the only significant covariate influencing CL in the final model. Inclusion of this covariate resulted in a marked improvement in the log-likelihood value of the base model. The typical population estimates for meropenem CL and Vc were 6.472 L/h and 26.69 L, respectively. The final covariate model was described as follows:

$$\text{CL}=6.472\times {\left(\frac{\text{CrCL}}{47.7}\right)}^{0.3834}\times \exp\left(\eta_{\text{CL}}\right)$$

$$\text{Vc}=26.69\times \exp\left(\eta_{\text{Vc}}\right)$$

CL and Vc represent meropenem clearance and apparent volume of distribution, respectively; CRCL denotes endogenous creatinine clearance calculated using the Cockcroft–Gault equation; and ηCL and ηVc represent interindividual variability for CL and Vc.

### 3.3 Model evaluation

**FIGURE 1:**
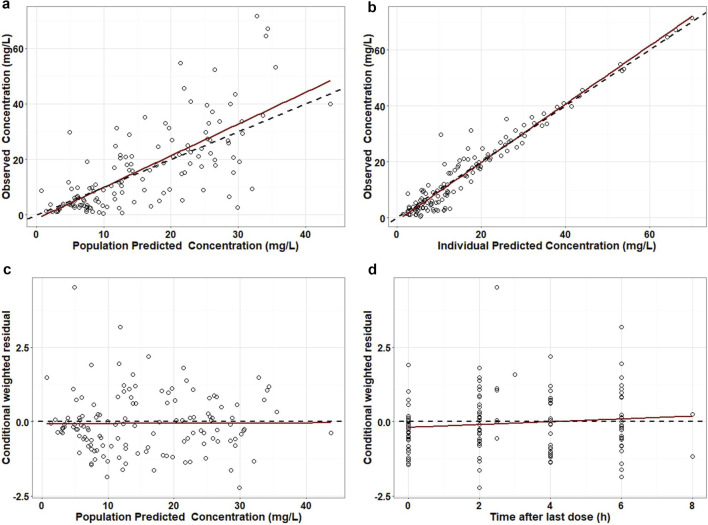
Diagnostic goodness-of-fit plots for the final population pharmacokinetic model. Panels show observed concentrations versus population-predicted concentrations **(a)** and individual-predicted concentrations **(b)** CWRES versus population-predicted concentrations **(c)** and CWRES versus time after dose **(d)**.

**FIGURE 2:**
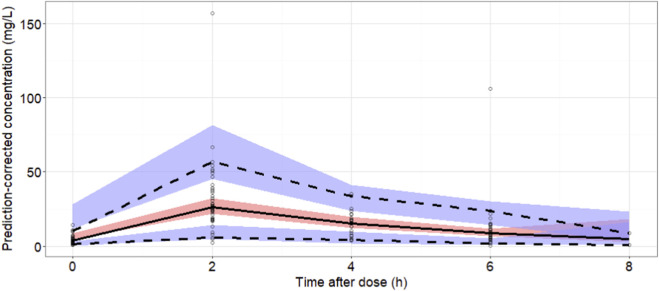
The pcVPCs of meropenem concentrations for the final population pharmacokinetic model over a single dosing interval. The solid black line represents the median (50th percentile) of the simulated concentrations, while the dashed lines indicate the 5th and 95th percentiles. Shaded areas denote the 90% confidence intervals for the corresponding simulated percentiles.

Diagnostic plots for the final model are presented in Figure 1. Both population-predicted and individual-predicted concentrations were symmetrically distributed around the line of identity (y = x), indicating good agreement between observed and predicted values. Most CWRES were evenly distributed between ±2 and centered around zero. In addition, the absolute CWRES trends were nearly parallel to the zero line, suggesting minimal systematic bias and an adequate model fit. The median parameter estimates and corresponding 95% confidence intervals obtained from 500 bootstrap datasets are shown in Table 2. The bootstrap-derived parameter medians were highly consistent with the final model estimates, and all final parameter values fell within the 95% confidence intervals. Notably, none of the bootstrap-derived confidence intervals included zero or negative values, supporting the robustness and stability of the final model. The results of pcVPCs are shown in Figure 2. The majority of observed concentrations fell within the 90% prediction interval, indicating satisfactory predictive performance of the model.

**TABLE 2:** Comparison of parameter estimates between the final model and bootstrap results.

		Final model		Bootstrap (N = 500)*	
OFV		Estimation		Median	95% CI
		685.07		673	603–733
Parameter	Unit	Typical value	RSE (%)	Median	95% CI
Fixed effect					
CL	L/h	6.472	8.53	6.46	5.52–7.81
Vc	L	26.69	13.55	26.6	20.3–38.1
CRCL on CL	—	0.3834	16.54	0.396	0.254–0.545
Random effect					
$\omega_{\text{CL}}$	%	49.4	16.46	51.9	35.9–75.4
$\omega_{V_{c}}$	%	81.99	17.63	99.2	58.8–199.0
$\omega_{CL*Vc}$	—	ρ = 0.94	16.12	ρ = 0.948	0.888–0.994
Residual error					
Additional error	mg/L	17.4	12.0	4.64	2.99–6.34

CRCL on CL, the influence coefficient of CRCL on CL; CL, central compartment clearance; Vc, central compartment volume; $\omega_{\text{CL}}$ , square root of inter-individual variance for CL; $\omega_{V_{c}}$ , square root of inter-individual variance for Vc; CI, confidence interval; $\omega_{CL*Vc}$ , covariance between interindividual variability of central volume and clearance; ρ, correlation of interindividual variability of central volume and intercompartmental clearance.

### 3.4 Monte Carlo simulation

**FIGURE 3:**
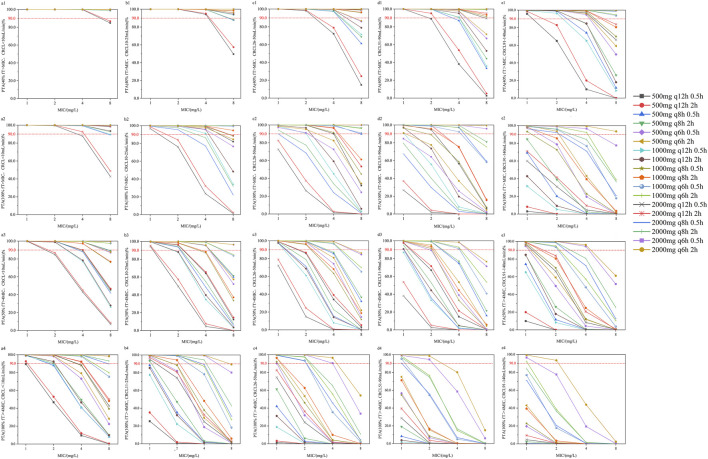
The PTAs of meropenem under different dosing regimens across varying MIC values, CRCL categories, and PK/PD targets. **(a1–e1,a2–e2,a3–e3,a4–e4)** correspond to PK/PD targets of 40% fT > MIC, 100% fT > MIC, 50% fT > 4×MIC,and 100% fT > 4×MIC,respectively. Panels **(a1–a4,b1–b4,c1–c4,d1–d4,e1–e4)** represent CRCL strata of <10 mL/min, 10–25 mL/min, 26–50 mL/min, 51–90 mL/min,and 91–140 mL/min,respectively.

Monte Carlo simulations were performed to estimate PTA for various dosing regimens across different renal function categories and MIC values. The simulation results are summarized in Figure 3 and Table 3. As shown in the simulations, 50% fT > 4×MIC was the most stringent PK/PD target compared with 100% fT > MIC and 40% fT > MIC, requiring substantially higher meropenem doses to achieve a PTA ≥90%. With increasing MIC values, doses far exceeding conventional recommendations were often required. For example, as illustrated in Figures 3b1–b4 and Table 3, when the PK/PD target was set to 40% fT > MIC with an MIC of 4 mg/L and a CRCL of 10–25 mL/min, all evaluated dosing regimens achieved a PTA ≥90%. However, when the target was increased to 100% fT > MIC, the regimen needed to be adjusted to 500 mg q8h administered over 30 min or 2 h. When targeting 50% fT > 4×MIC or 100% fT > 4×MIC, daily doses of 3 g or 6 g were required, which substantially exceed standard dosing recommendations. Although meropenem is generally well tolerated in clinical practice and dose adjustment is often unnecessary in patients with mild renal impairment, Monte Carlo simulations indicated that when bacterial susceptibility was high (MIC = 1 mg/L), a reduced dose of 500 mg q8h was sufficient to achieve all three PK/PD targets. To attain the stringent 50% fT > 4×MIC target, however, prolongation of the infusion duration to 2 h was required. These findings suggest that, in selected patients, dose reduction combined with prolonged infusion may be a viable strategy to reduce overall antibiotic exposure and potentially limit the development of resistance (Figures 3d1–d3; Table 3). When bacterial susceptibility was lower (MIC = 4 mg/L), simulations recommended increasing the single dose to 2 g to achieve the 50% fT > 4×MIC and 100% fT > MIC targets (Figures 3d2–d3; Table 3). But none of the simulated monotherapy regimens achieved the PK/PD target of 100% T > 4×MIC (Figure 3d4; Table 3). In cases involving multidrug-resistant pathogens (MIC = 8 mg/L), even the most aggressive regimen evaluated (2 g q6h) failed to achieve a PTA≥90% for the 50% fT > 4×MIC target, indicating the need for combination therapy or alternative antimicrobial agents (Figure 3d3; Table 3). Notably, in patients with severe renal impairment (CRCL <10 mL/min), prolonged infusion resulted in more stable plasma concentrations and improved safety and efficacy, particularly when pathogens exhibited reduced susceptibility (Figure 3a2; Table 3). When the total daily dose of meropenem was equivalent, increasing dosing frequency appeared to be more effective than increasing the single dose. For patients with moderate to severe renal impairment (CRCL 10–25 mL/min), regimens of 500 mg q6h or 500 mg q8h achieved higher PTA values and target attainment rates compared with 1,000 mg q12h, as shown in Figures 3b1–b3.

**TABLE 3:** Recommended meropenem dosing regimens to achieve PTA ≥90% based on Monte Carlo simulation.

MIC (mg/L)	CRCL				
	<10 mL/min	10–25 mL/min	26–50 mL/min	51–90 mL/min	91–140 mL/min
(1) PK/PD target: 40% fT > MIC					
1	All	All	All	All	All
2	All	All	All	500 mg q8h	500 mg q8h
4	All	All	500 mg q8h	500 mg q8h (2 h) or 1,000 mg q12 h	500 mg q6h
8	500 mg q8h	500 mg q6h	1,000 mg q8h	1,000 mg q8h	1,000 mg q6h
(2) PK/PD target: 100% fT > MIC					
1	All	All	500 mg q8h	500 mg q8h	500 mg q6h
2	All	500 mg q8h	500 mg q8h	500–2,000 mg q6h	500 mg q6h (2 h)
4	500 mg q12 h (2 h)	500 mg q8h	500 mg q6h	1,000–2,000 mg q6h or 2000 mg q8h	1,000 mg q6h (2 h)
8	500 mg q8h or 1,000 mg q12 h (2 h)	1,000 mg q8h (2 h)	1,000–2,000 mg q6h	2,000 mg q6h	Only 2,000 mg q6h (2 h)
(3) PK/PD target: 50% fT > 4 × MIC					
1	All	All	500 mg q8h	500 mg q8h (2 h)	500 mg q6h
2	500 mg q8h	500 mg q6h	1,000 mg q8h	1,000 mg q8h	1,000 mg q6h
4	1,000 mg q8h	1,000 mg q6h or 2,000 mg q12 h	2,000 mg q6–8 h	2,000 mg q6–8 h	Only 2,000 mg q6h
8	2,000 mg q6–8 h	2,000 mg q6h	None	None	None
(4) PK/PD target: 100% fT > 4 × MIC					
1	500 mg q12 h (2 h)	500 mg q8h (2 h)	500 mg q6h	1,000 mg q6h	2,000 mg q6h
2	500 mg q8h (2 h)	1,000 mg q8h (2 h)	1,000 mg q6h	2,000 mg q6h	Only 2,000 mg q6h (2 h)
4	1,000 mg q8h (2 h)	2,000 mg q8h (2 h)	2,000 mg q6h	None	None
8	2,000 mg q8h (2 h)	None	None	None	None

(1) Dosing regimens were evaluated by Monte Carlo simulation and included meropenem doses of 500, 1,000, and 2,000 mg administered every 6, 8, or 12 h, with infusion durations of 0.5 or 2 h.

(2) T > MIC, and T > 4×MIC, denote the percentage of the dosing interval during which free drug concentrations exceed the MIC, or fourfold MIC, respectively.

(3) CRCL, represents creatinine clearance calculated using the Cockcroft–Gault equation.

(4) MIC, values were based on the 2025 CLSI, susceptibility breakpoints for Enterobacterales, *Pseudomonas aeruginosa*, and *Acinetobacter* baumannii.

(5) “(2 h)”indicates that infusion duration was 2h, “All” indicates that all evaluated regimens achieved PTA ≥90%, whereas “None” indicates that no regimen met the target.

## 4 Discussion

In this prospective therapeutic drug monitoring cohort of adults with severe postoperative infections, we developed a population pharmacokinetic model for meropenem and used Monte Carlo simulations to support renal function stratified dosing under clinically relevant PK/PD targets. A one-compartment model with linear elimination adequately described the concentration–time data from 44 patients with 135 samples. This structural model is consistent with several previous studies, although many population pharmacokinetic investigations of meropenem have also employed a two-compartment model (Jaruratanasirikul et al., 2015; Murínová et al., 2024; O'Jeanson et al., 2021; Gijsen et al., 2022; Lan et al., 2022; Boonpeng et al., 2022). Model diagnostics suggested acceptable performance: observed concentrations were reasonably consistent with both population and individual predictions, residuals were centered around zero without obvious trends, and the visual predictive check showed that most observations fell within the simulated prediction intervals. Bootstrap analyses further supported parameter stability.

The typical population estimates of CL and Vc in our study were 6.472 L/h and 26.69 L, respectively. A key finding was that postoperative creatinine clearance was the only significant covariate influencing meropenem clearance. The estimated CL was lower than that reported in postoperative critically ill patients in Pakistan (12.2 L/h) but was comparable to values observed in septic ICU populations (7.82 L/h), elderly patients (5.27 L/h), and patients undergoing sustained low-efficiency dialysis (7.9 L/h) (Jaruratanasirikul et al., 2015; Usma et al., 2017; Braune et al., 2018). These differences may be attributed to demographic and clinical heterogeneity, including the older age, lower body weight, reduced creatinine clearance, and the inclusion of non-ICU patients in our cohort. In addition, the presence of renal impairment and hemodialysis in a subset of patients likely contributed to the lower overall clearance estimate. Collectively, these findings suggest that postoperative status itself may not independently increase meropenem clearance beyond the influence mediated by renal function. The typical parameter estimates were clinically plausible, yet interindividual variability remained substantial, emphasizing that fixed dosing may not reliably achieve adequate exposure in postoperative patients. Because renal function can change rapidly after surgery due to hemodynamic fluctuations, inflammatory responses, and shifts in volume status, frequent reassessment and timely dose adjustment are essential. These results provide a pharmacokinetic rationale for combining dynamic renal monitoring with TDM guided individualization, particularly early after surgery when underexposure may compromise efficacy and overexposure may increase toxicity risk.

In current clinical practice, the standard meropenem regimen for severe infections is 1 g every 8 h, with dose escalation to 2 g every 8 h or 2 g every 6 h in selected critically ill patients (Tamma et al., 2024). Our simulation results translated these findings into clinically relevant dosing strategies across different renal function strata and MIC scenarios. As expected for a hydrophilic, time-dependent β-lactam antibiotic, conventional PK/PD targets such as 40% fT > MIC were generally achieved at lower MIC values with standard dosing regimens. In contrast, stricter PK/PD targets including 100% fT > MIC, and particularly 50% fT > 4×MIC and 100% fT > 4×MIC required intensified dosing strategies with increasing MIC. For highly susceptible pathogens (MIC 1 mg/L), most regimens achieved high PTA across all renal function groups, and prolonged infusion enhanced robustness for stringent targets without increasing the total daily dose. As the MIC increased to 4 mg/L, PTA was reduced unless dosing frequency was intensified, infusion was prolonged, or both, supporting strategies that increase time above MIC rather than relying merely on larger single doses. At an MIC of 8 mg/L, simulation results showed that even intensive dosing regimens were unable to attain stringent PK/PD targets in multiple renal function strata. This indicates distinct limitations to meropenem intensification and underscores the importance of considering alternative active antimicrobials or combination strategies in the setting of suspected or confirmed reduced susceptibility. For regimens with similar total daily doses, more frequent administration generally yielded higher PTA than escalating single doses, whereas prolonged infusion consistently favored the attainment of time-based PK/PD targets. These observations are consistent with several studies using TDM–guided individualized dosing, which have demonstrated that extended infusion, increased dosing frequency, or higher single doses may improve therapeutic exposure (Fournier et al., 2015; Shi et al., 2023).

The 2024 IDSA guidance recommends extended infusion as the preferred administration strategy for infections caused by resistant Gram-negative pathogens, including carbapenem-resistant Enterobacterales (CRE), difficult-to-treat resistant *Pseudomonas aeruginosa* (DTR-PA), and carbapenem-resistant *Acinetobacter* baumannii (CRAB), particularly in critically ill or ICU patients and in infections caused by organisms with elevated MIC values (Tamma et al., 2024). Although continuous intravenous infusion can theoretically maximize %T > MIC, its clinical application is often limited by drug stability and practical considerations, and it is generally considered only when extended infusion fails to achieve PK/PD targets. In our simulations, prolonged infusion substantially improved target attainment and represents a pragmatic balance between pharmacodynamic optimization and clinical feasibility. For pathogens with markedly elevated MIC values, however, even continuous infusion may not fully overcome the intrinsic exposure limitations of meropenem monotherapy. Although meropenem generally has a favorable safety profile, the risk of neurotoxicity and nephrotoxicity increases at elevated plasma concentrations, with reported trough concentration thresholds of 64.2 mg/L and 44.45 mg/L, respectively (Beumier et al., 2015; Imani et al., 2017). This risk is particularly pronounced in patients with renal impairment. In the present study, although a small number of patients exhibited trough concentrations exceeding these toxicity thresholds, none developed clinically apparent neurotoxicity or nephrotoxicity. This observation may be related to the limited number of cases with markedly elevated drug exposure. Therefore, intensified dosing regimens should be used with caution and are ideally guided by therapeutic drug monitoring, with further confirmation in prospective studies.

### 4.1 Limitations of the study

Several limitations should be noted. This was a single center study with a modest sample size, which may limit detection of additional covariates and generalizability. Individual microbiology and MIC data were incomplete, so MIC scenarios were used for empiric decision making but cannot replace susceptibility guided optimization. In addition, we evaluated PK/PD target attainment rather than directly linking exposure to clinical outcomes. Future multicenter studies incorporating comprehensive microbiology, external validation, and outcome endpoints are warranted, particularly in patients with augmented renal clearance, fluctuating renal function, or renal replacement therapy.

## 5 Conclusion

Overall, our findings show that postoperative renal function is the principal driver of meropenem exposure variability and support model informed, renal stratified dosing that prioritizes dosing frequency and infusion optimization to improve target attainment while recognizing the boundary of meropenem optimization in high MIC settings.

## Data Availability

The original contributions presented in the study are included in the article/supplementary material, further inquiries can be directed to the corresponding authors.
